# Simulation and minimization: technical advances for factorial experiments designed to optimize clinical interventions

**DOI:** 10.1186/s12874-019-0883-9

**Published:** 2019-12-16

**Authors:** Jocelyn Kuhn, Radley Christopher Sheldrick, Sarabeth Broder-Fingert, Andrea Chu, Lisa Fortuna, Megan Jordan, Dana Rubin, Emily Feinberg

**Affiliations:** 1 Boston Medical Center, 72 E. Concord St, Boston, MA USA; 2 Boston University School of Public Health, 801 Massachusetts Ave, Boston, MA USA; 3 Boston University School of Medicine, 72 E. Concord St, Boston, MA USA; 4 DotHouse Health Center, 1353 Dorchester Ave, Dorchester, MA USA

**Keywords:** Clinical trials, Clinical intervention studies, Randomization, Subject allocation, Minimization, Factorial design, Multi-phase optimization strategy

## Abstract

**Background:**

The Multiphase Optimization Strategy (MOST) is designed to maximize the impact of clinical healthcare interventions, which are typically multicomponent and increasingly complex. MOST often relies on factorial experiments to identify which components of an intervention are most effective, efficient, and scalable. When assigning participants to conditions in factorial experiments, researchers must be careful to select the assignment procedure that will result in balanced sample sizes and equivalence of covariates across conditions while maintaining unpredictability.

**Methods:**

In the context of a MOST optimization trial with a 2x2x2x2 factorial design, we used computer simulation to empirically test five subject allocation procedures: simple randomization, stratified randomization with permuted blocks, maximum tolerated imbalance (MTI), minimal sufficient balance (MSB), and minimization. We compared these methods across the 16 study cells with respect to sample size balance, equivalence on key covariates, and unpredictability. Leveraging an existing dataset to compare these procedures, we conducted 250 computerized simulations using bootstrap samples of 304 participants.

**Results:**

Simple randomization, the most unpredictable procedure, generated poor sample balance and equivalence of covariates across the 16 study cells. Stratified randomization with permuted blocks performed well on stratified variables but resulted in poor equivalence on other covariates and poor balance. MTI, MSB, and minimization had higher complexity and cost. MTI resulted in balance close to pre-specified thresholds and a higher degree of unpredictability, but poor equivalence of covariates. MSB had 19.7% deterministic allocations, poor sample balance and improved equivalence on only a few covariates. Minimization was most successful in achieving balanced sample sizes and equivalence across a large number of covariates, but resulted in 34% deterministic allocations. Small differences in proportion of correct guesses were found across the procedures.

**Conclusions:**

Based on the computer simulation results and priorities within the study context, minimization with a random element was selected for the planned research study. Minimization with a random element, as well as computer simulation to make an informed randomization procedure choice, are utilized infrequently in randomized experiments but represent important technical advances that researchers implementing multi-arm and factorial studies should consider.

## Background

An emerging methodology known as the Multiphase Optimization Strategy (MOST), which was inspired by engineering frameworks and guides research questions related to identifying the *optimal* version of an intervention, is receiving increasing attention in the healthcare field. The MOST framework includes three phases: *preparation* of a conceptual model with identified intervention components that impact the intervention effectiveness, *optimization* of the intervention with a trial that evaluates the performance of the individual intervention components, and *evaluation* of the optimized intervention with a randomized controlled trial (RCT). Unlike the traditional RCT framework which compares a treatment group that receives an intervention package to a control group, the MOST framework tests the anticipated “active ingredients” (i.e., intervention components), thus providing results on the most effective, efficient, and scalable form of an intervention [1, 2].

MOST *optimization* trials utilize factorial experimental design [3–5] because they can test multiple factors (i.e., intervention components or delivery strategies) simultaneously, using the same participants while maintaining satisfactory statistical power [6]. For example, a factorial design with two intervention components with two levels each yields four *cells* (i.e., 2 × 2 = 4), each representing a group of participants assigned to a study condition that receives a unique combination of intervention component levels. As the number of intervention components in the factorial design increases, the number of cells grows exponentially (i.e., four components with two levels each requires 2 × 2 × 2 × 2 = 16 cells). Because participants in factorial experiments are independently assigned to a level on each factor and factors are analyzed separately for main effects, statistical power will generally be equivalent to a single-factor RCT that has the same number of study arms as the factorial design’s number of levels within each factor.

Despite the benefits that factorial designs offer with regard to sample size and statistical power, they also present complexity and challenges for subject allocation, especially when the number of cells is large. [7] Consensus guidelines for reporting randomized trials (i.e., Consolidated Standards of Reporting Trials (CONSORT) [8]) describe a range of acceptable methods for allocation of participants to study cells and suggest that three criteria are important for determining which method to use. First, participant allocation should result in *balanced sample sizes* across study conditions to maximize statistical power [8–10]. Second, participant allocation should result in study conditions that are equivalent with respect to covariates that are expected to impact intervention outcomes (i.e., *equivalent groups*) [11]. Third, participant allocation should be completely unpredictable to both study staff and to participants so as to ensure that measured and unmeasured participant characteristics, and selection biases in general, do not influence participants’ assignment to conditions. Given the large number of cells in factorial experiments and division of participants across those cells, balanced sample sizes and equivalent groups are especially important, yet may be difficult to achieve. Finally, we suggest additional criteria that are common to many practical decisions: cost and complexity including resource utilization. Some allocation procedures can be readily implemented using a range of accessible methods and software, whereas other methods may require coding or specialized software that must then be incorporated into workflow. The four outcomes of interest in the present study are these four criteria for subject assignment methods: balance of sample size, equivalence of groups, unpredictability, and low complexity.

The CONSORT statement [8] classifies the range of acceptable assignment methods into three categories: *Simple randomization,* which includes the use of random number tables, computerized random number generators, or even a coin toss. *Restricted randomization* involves combining random assignment with additional strategies to improve balance and equivalence across cells. For example, assigning participants in *blocks* that are the same size as (or a multiple of) the number of cells promotes balanced sample size across conditions [8, 10]. *Stratification* defines subsets of participants within which random assignment with blocking occurs [8], thus promoting equivalence in the baseline characteristics used to define the strata [12].

Lastly, adaptive randomization procedures show advantages over more traditional restrictive and simple randomization procedures [13]. *Maximum tolerated imbalance (MTI)* represents a class of more novel adaptive randomization procedures which defend against selection bias by implementing simple randomization until a pre-defined imbalance in sample sizes occurs, at which point a “big stick” is used to deterministically regain balanced sample sizes across conditions [14]. For cases in which equivalence of covariates is of utmost concern rather than balanced sample sizes, covariate adaptive randomization strategies such as *minimal sufficient balance (MSB)* can be used. MSB uses simple randomization until inequivalence on a covariate is reached, which is determined quantitatively by pre-specified *p-value* limits from *t-tests*; when the *p-value* limit is reached on a covariate across conditions, a more predictable assignment, such as biased coin assignment, is implemented to achieve equivalence once again [15, 16]. In contrast, *minimization* involves assigning each participant to the condition that minimizes differences in sample size and pre-specified covariates across the all of the study cells [8]. In other words, participants are allocated to the cell that would result in the minimum sum of ranges of both sample size and covariates if the participant were to be assigned to each possible cell. Although this appears fundamentally deterministic, a random element can be introduced to settle ties among study cells [17]. In the case of a factorial design with 16 cells, sample size and covariate ties become more common occurrences; randomized allocation between tied cells becomes a logical and necessary technique to incorporate into the minimization procedure.

As expected, each of these subject allocation methods theoretically has strengths and weaknesses with regard to balance, equivalence, unpredictability, and complexity (see Table 1). With regard to predictability, *simple randomization* is the most unpredictable on a theoretical level and is therefore best for reducing selection bias [10, 13]. In comparison, *restricted randomization* includes a variety of procedures, each with varying levels of selection bias threat. Blocking heightens selection bias because block size is generally known to study investigators, and assignment becomes increasingly predictable as cumulative enrollment reaches numbers equivalent to multiples of the number of study conditions [8, 18]. While introducing random variation in block size (i.e., permuted blocks) can mitigate this problem [8], the benefit of doing so with respect to balance declines as the number of study cells increases—as is often the case for factorial experiments. In contrast, adaptive MTI and MSB procedures minimize selection bias with the use of simple randomization up until implementation of a more deterministic method becomes necessary based on pre-determined limits [14, 16]. Thus, the degree of predictability of *restricted randomization* and *adaptive randomization* depends on the exact procedure used and the number of arms or factors in the study design to which it is applied.

**Table 1:** Theoretical comparison of allocation methods

	Unpredictability	Balanced sample sizes across conditions	Equivalent baseline characteristics across conditions	Cost & complexity
Simple randomization	Best; random assignment prevents predictability	Poor; likely to result in differences across cells	Poor; likely to result in differences across cells	Best; simple to implement
Stratification with permuted blocks	OK; Random order of assignments within blocks within strata reduces predictability but known block sizes increase predictability	Very good; blocking improves balance, but this is mitigated by stratification	Good; stratification improves equivalence on specific variables	Very good; more complex, but solutions are widely available
Maximum tolerated imbalance	Very good; random assignment protects against selection bias until big stick is needed.	Very good; results at or below maximum tolerated imbalance of samples	Poor; No better than simple randomization	OK; can be implemented in a range of available software, but requires coding
Minimal sufficient balance	Very good; random assignment protects against selection bias until biased coin is needed	Poor; No better than simple randomization	Very good; results at or below maximum tolerated inequivalence of covariates	OK; can be implemented in a range of available software, but requires coding
Minimization	Poor when purely deterministic; improved with incorporation of random element	Very good; should promote balance, depending on algorithm	Best; promotes equivalence on a large number of variables	OK; can be implemented in a range of available software, but requires coding

Conversely, *minimization* without a random element is an inherently deterministic procedure that can be predicted given perfect knowledge of prior assignments and covariate data for the next participant being assigned, as well as the algorithm by which these values result in assignment. The more covariates included in the minimization algorithm, the more difficult it would theoretically be for an investigator to keep track of such information mentally; nevertheless, selection bias remains a pitfall of this purely deterministic method, and even simple guessing rules may have the potential to exceed chance levels [19]. Adding elements of random assignment into minimization algorithms is preferable because such methods reduce reliance on deterministic allocations, and reduce the likelihood of an investigator’s guess of an assignment being correct [8]. Thus, for minimization, predictability again depends on the exact procedure used and the study design to which it is applied.

When seeking balanced sample sizes and groups that are equivalent with respect to baseline variables across study conditions, simple randomization is expected to perform the most poorly [10]. The credibility of factorial experiments can be significantly compromised by simple randomization because of the compounded problem of yielding cells that are imbalanced with respect to sample size and non-equivalent with respect to key covariates [8]. Many researchers continue to follow the precedent of using stratification with permuted blocks to address these issues; however, making such determinations based on precedent can be misguided and ignores the substantial threat of selection bias that blocking creates [20, 21]. An additional limitation to stratification with permuted blocks is that when block size is equivalent to the number of study conditions, stratification is only feasible for two or three variables at most [12]. In the context of factorial designs, stratification with permuted blocks is therefore additionally limited in the number of variables on which it can promote equivalence. MTI and MSB methods protect from selection bias with a default to simple randomization, but only up until the limit on tolerated imbalance or inequivalence is reached. MTI satisfies the need for a pre-determined level of balance on sample size, whereas MSB supports equivalence on selected covariates. In factorial design studies requiring both balance and equivalence, implementing one of these techniques alone will not be sufficient.

*Minimization* procedures can ensure that conditions have balanced sample sizes and equivalent baseline characteristics for a large number of variables, even for studies with small sample sizes and/or many treatment conditions, across all stages of an experiment. Some therefore argue that *minimization* procedures are not only acceptable, but a superior alternative to simple or restricted randomization techniques such as stratification with permuted blocks [22]. In *minimization* assignment, the first patient is assigned at random, and each following participant is assigned to the condition that minimizes differences across study conditions with respect to sample size and selected covariates. Assignment becomes less easily guessed correctly by researchers as more variables are added and the minimization algorithm becomes more complex [23]. Moreover, researchers may incorporate randomization into their minimization scheme. For study designs with more than two treatment conditions, randomization may also be necessary if the minimization algorithm results in ties among two or more conditions. Researchers may even choose to set up the minimization algorithm to incorporate randomization for near-ties or to use a weighted probability that favors, but does not determine, assignment to the condition that minimizes imbalances [8]. Such methods appear to be effective mechanisms to reduce the risk of minimization assignment from being fully “deterministic” [17].

Finally, assignment strategies differ with respect to cost and complexity. In this regard, simple randomization is arguably best as a wide range of software and even a coin or die can be sufficient. Stratification with permuted blocks is a close second because it is embedded in a range of software packages used by researchers, including RedCap. While simple conceptually, MTI, MSB, and minimization procedures are currently the most difficult to implement, requiring specialized coding in software packages like Excel, Stata, or R. These randomization algorithms must be individualized based on covariates of interest, thresholds of acceptable imbalance or inequivalence, and number of arms in particular studies. Once the minimization and MSB programs are written, study staff must obtain sufficient covariate data (e.g., age, ethnic identity, co-morbid conditions) before randomizing participants to run the program. MTI, MSB, and minimization programs will likely be stand-alone as none of these procedures are currently integrated into other commonly used research study management systems such as RedCap or StudyTrax. Overall, MTI, MSB, and minimization are the most complex because they require additional skills, staff time, and resources.

### Aims of the current study

Given the relative strengths and weakness of simple randomization, stratification with permuted blocks, MTI, MSB, and minimization procedures for participant assignment with respect to a given study design (e.g., number of cells, sample size) and hypotheses (e.g., number of conditions of interest, importance of testing interactions), it is critical that researchers make deliberate, informed choices about their participant assignment procedures for their unique studies, particularly in the context of factorial designs within MOST frameworks. The primary aim of this paper is to present a case study of how assignment procedures can be directly compared by conducting simulations drawing from a prior locally-collected dataset.

## Method

In this paper we describe a process for selecting among participant assignment procedures. We conducted a series of simulations to inform our participant assignment procedure selection for a MOST *optimization* study designed to determine how to best deliver an evidence-based care coordination strategy called Family Navigation [24] within the context of child mental health. Specifically, this *optimization* study uses a 2x2x2x2 factorial design to test the impact of each of four delivery strategies: a) technology-assisted vs. traditional care management; (b) community vs. clinic-based delivery; (c) enhanced vs. routine symptom tracking; and (d) fixed vs. flexible schedule of visits. Based on a priori power analysis, we intend to enroll a sample of 304 families.

We adopted a three-stage method to determine the optimal assignment strategy: [1] review literature and define assignment procedures [2]; conduct simulations on a dataset from a previous study, reporting outcomes for each strategy with respect to balance, equivalence, and unpredictability (i.e., proportion of deterministic assignments, average number of potential conditions across allocations, and correct guesses) [3]; review results with the research team; determine which method is optimal in the study context across all outcomes.

Step 1: Based on our review of the literature, we operationalized each assignment method. *Simple randomization* was determined using the random number generator in Stata version 15. We implemented two maximum tolerated imbalance (*MTI)* procedures: one with a pre-specified MTI of 2 (MTI2), and one with a pre-specified MTI of 3 (MTI3). For these procedures, participants were assigned at random unless the difference between the minimum and maximum cell size across the sixteen cells exceeded the pre-specified values of 2 or 3, at which point a big stick was used to assign the participant to the cell with the smallest sample size (with ties resolved by random assignment). Modified to accommodate allocations to sixteen possible cells, our implementation of MSB also assigned participants at random, but a different algorithm triggered more directed assignments. Before each allocation, chi-square tests were conducted for each of eight binary covariates (Medicaid status, work outside home, sex, Hispanic (yes/no), Black (yes/no), child age (old/young), autism diagnosis (yes/no), high school education (yes/no), and married or living with partner (yes/no)) to test for imbalance. Votes were assigned if imbalance was statistically significant at an alpha < 0.30 level. If the next participant was positive with respect to the given covariate (i.e., Hispanic = 1), then a “vote” was assigned to the cell with the lowest proportion with respect to that covariate. If the next participant was negative on the given covariate (i.e., Hispanic = 0), then a “vote” was taken away from the cell with the highest proportion with respect to that covariate. After testing all covariates, the participant was assigned to the cell with the highest votes, and ties were resolved by random assignment.

*Stratification with Permuted Blocks* was conducted within permuted blocks of 16 and 32 participants within three strata defined by race/ethnicity (coded as: 1. Hispanic, 2. non-Hispanic Black, or 3. other). *Minimization* was conducted based on eight binary covariates (Medicaid status (Medicaid/other), work outside home (yes/no), sex (male/female), Hispanic (yes/no), Black (yes/no), child age (old/young), autism diagnosis (yes/no), high school education (yes/no), and married or living with partner (yes/no), which were selected based on their availability as binary socio-demographic variables that our investigator team believed were necessary to evenly represent across cells. The minimization algorithm calculated the sum of ranges based on cell sizes and covariates that would result if the next subject were allocated to each possible cell, and then made the next assignment that minimized the sum of ranges. In cases when more than one cell shared the same minimum sum of ranges, ties were determined with simple random assignment (i.e., 1:2 chance of assignment with a tie of 2, 1:3 chance of assignment with a tie between 3 cells).

Step 2: We selected an existing dataset from a recent previous study by our group that took place in the same pediatric clinic population and urban area. The existing dataset contained 332 participants. We conducted 250 simulations that directly compared the three assignment procedures. For each simulation, we drew a bootstrap sample of 304 participants, which was the planned sample size for the proposed study. This sample of participants was then assigned to study cells using each of the methods described in Step 1. We measured the performance of each technique based on balance, equivalence, and predictability.

To assess balance, we calculated the following statistics for each of the 250 simulations: mean minimum cell size, mean maximum cell size, and mean range (i.e., difference between minimum and maximum cell sizes) of the 250 mean cell sizes. In the context of a 16-cell factorial design study with 304 participants, which would ideally have 19 participants in each cell, we determined a mean sample size range of less than or equal to two as acceptable, and that anything higher would be problematic with regard to statistical power. To assess equivalence, we conducted statistical tests for differences with respect to each covariate and reported the average proportion of covariates that displayed statistically significant differences at the end of each simulation.

Following previous literature and with consideration of the unique nature of factorial experiments, we used several metrics to assess unpredictability. First, we tracked the number of eligible cells for each allocation, ranging from 1 to 16. Each time a participant is allocated to a combination of the four treatment conditions (i.e., a study cell), anywhere between 1 and 16 cells are eligible for assignment. If only one cell is eligible, the allocation is deterministic. If all 16 cells are eligible and all have equal weights, then the allocation is completely random. Numbers between 2 and 15 represent allocations that are neither completely random nor fully deterministic.

In addition, past research suggests that even simple guessing rules can yield predictions that substantially exceed chance [25]. Following previous literature [25, 26], we assessed the success of a guess based on selecting the cell with the minimum sample size (hereafter, “Correct Guess 1”). We also assessed additional guessing rules to address the special nature of factorial designs. For example, a researcher could potentially wish to influence assignment to a particular factor, for example by guessing that the next participant will be assigned to the level of a factor with the smallest overall sample size (hereafter, “Correct Guess 2”). Knowing that minimization and MSB allocation algorithms are designed to maximize equivalence, a research staff member may also incorporate knowledge of covariates. Therefore, we included a third guessing rule that assigned a point to each level of a factor if it: (a) had the smaller sample size, and (b) included a smaller number of Black children. Guesses were assigned to the factor with the greater number of points and ties were resolved by random selection (hereafter, “Correct Guess 3”).

Step 3: The research team reviewed simulation results while also considering feasibility and cost of implementation. A priori, we intended to prioritize outcomes in the following order: unpredictability, balance, equivalence on stratification variables, and equivalence on other covariates. Based on the research team’s experiences on prior studies that implemented stratification with permuted blocks for randomization, this was our default choice. We anticipated that an alternative procedure would need to demonstrate substantial improvements in terms of balance and equivalence to justify their increased complexity.

## Results

Table 2 displays simulation results with respect to balanced sample size across cells. As expected, simple randomization and MSB performed poorly with average difference between minimum and maximum cell sample sizes of 15.3 and 9.2, respectively. Stratification with permuted blocks was similar to MSB, with an average difference in 9.1 participants between the largest and smallest cell sizes. As expected, the MTI procedure led to improved balance, with cell size ranges hovering closely around the pre-determined maximum tolerated imbalance values. Minimization resulted in the best balance, with an average cell size difference of only 1.9. Note that with a planned sample size of 304, the optimal sample size was 19 participants per cell (and a range of cell sizes of 1 was not mathematically possible).

**Table 2:** Sample size balance: Differences in 16 cell sizes across 250 simulations

	Min	Max	Range
Simple randomization	11.8	27.1	15.3
Stratification with permuted blocks	14.5	23.6	9.1
MTI2	18	20.9	2.9
MTI3	18	21.4	3.4
MSB	15.2	24.4	9.2
Minimization	18.0	19.9	1.9

*Note.* Min = Mean minimum cell size, Max = Mean maximum cell size

Table 3 displays simulation results regarding equivalence on the two binary stratification covariates (SCs) that were related to strata in the stratification with permuted blocks procedure, as well as the other six binary minimization covariates (MCs). As expected, minimization performed best, with no significantly different covariates across cells in all 250 simulations. Stratification with permuted blocks performed well on the two SCs, but had an average of 30.8% of the additional minimization covariates with statistically significant differences across cells. MSB also performed relatively well on the two strata-related covariates, but did not scale well to sixteen cells with eight covariates, resulting in an average of 37.8% of the MCs with a statistically significant difference. Simple randomization and the MTI procedures performed worst across both SCs and MCs.

**Table 3:** Number of stratification and minimization covariates with a statistically significant difference across cells in 250 simulations

	Number of SCs with statistically significant differences			Ave. % of SCs	Number of MCs with statistically significant differences							Ave. % of MCs
	0	1	2		0	1	2	3	4	5	6	
SR	92.4%	7.2%	0.4%	8.0%	67.6%	27.6%	4.8%	0.0%	0.0%	0.0%	0.0%	37.2%
SPB	99.6%	0.0%	0.4%	0.8%	72.4%	24.4%	3.2%	0.0%	0.0%	0.0%	0.0%	30.8%
MTI2	91.2%	7.2%	1.6%	10.4%	68.4%	24.4%	7.2%	0.0%	0.0%	0.0%	0.0%	38.8%
MTI3	92.0%	6.4%	1.6%	9.6%	66.4%	24.8%	8.0%	0.8%	0.0%	0.0%	0.0%	43.2%
MSB	95.6%	4.0%	0.4%	4.8%	69.2%	26.0%	3.6%	0.8%	0.4%	0.0%	0.0%	37.2%
Min.	100.0%	0.0%	0.0%	0.0%	100.0%	0.0%	0.0%	0.0%	0.0%	0.0%	0.0%	0.0%

*Note.* SR = simple randomization; SPB = stratification with permuted blocks; MTI = maximum tolerated imbalance; MSB = minimal sufficient balance; Min. = minimization; SC = stratification covariate; MC = minimization covariate

With regard to unpredictability, simple randomization was best by definition, with 100% random assignment to one of 16 cells. For stratification with blocking, blocks of 16 and 32 participants suggest that 15/16 = 94% and 31/32 = 96.9% involved at least some degree of random assignment depending on block size. Results were consistent with this, with 4% of deterministic guesses to one cell. For MTI2 and MTI3, 5.9% of assignments were deterministic, requiring use of the big stick. MSB and minimization procedures performed worst in terms of proportion of deterministic assignments, with 20 and 34% deterministic assignments, respectively Table 4.

**Table 4:** Proportion of allocations that are deterministic, fully random, or in between

Cells																	
	1	2	3	4	5	6	7	8	9	10	11	12	13	14	15	16	Ave. cells
SR	0.0%	0.0%	0.0%	0.0%	0.0%	0.0%	0.0%	0.0%	0.0%	0.0%	0.0%	0.0%	0.0%	0.0%	0.0%	100%	16.0
SPB	4.2%	4.3%	4.5%	4.5%	4.6%	4.7%	4.9%	5.1%	5.4%	5.4%	5.6%	6.2%	6.9%	7.4%	9.9%	18.2%	10.4
MTI2	5.9%	5.9%	5.9%	5.8%	5.4%	5.0%	4.4%	3.6%	2.9%	2.1%	1.5%	1.0%	0.6%	0.3%	0.1%	49.7%	10.6
MTI3	5.9%	5.7%	5.3%	4.8%	4.2%	3.5%	2.8%	2.1%	1.5%	1.1%	0.7%	0.4%	0.2%	0.1%	0.0%	61.6%	11.6
MSB	19.7%	16.1%	13.2%	10.8%	9.0%	7.4%	5.9%	4.4%	3.0%	2.0%	1.3%	1.1%	0.8%	0.8%	0.6%	3.9%	4.7
Min	34.0%	18.8%	13.7%	10.0%	7.1%	4.6%	2.8%	1.7%	0.9%	0.5%	0.3%	0.1%	0.1%	0.0%	0.0%	0.0%	2.7

*Note.* 1 cell = deterministic; 16 cells = completely random. SR = simple randomization; SPB = stratification with permuted blocks; MTI = maximum tolerated imbalance; MSB = minimal sufficient balance; Min = minimization. Ave. cells = average number of cells eligible for randomization per allocation

Regarding correct guess based on knowledge of smallest cell size (Correct Guess 1), MTI with a pre-specified value of 2 performed the most poorly, with a 19.2% chance of guessing an assignment to an exact cell correctly. The MTI3, Minimization, and MSB procedure resulted in only slightly better guessing results, ranging between 18.2 and 15.5%. Stratification with permuted blocks and simple randomization performed best with the lowest proportion of correct guesses to a single cell. Given knowledge of the factor level with the smallest combined cell sizes (Correct Guess 2), results across procedures ranged between 50 and 60%, with simple randomization performing best (50.1%) and MTI2 performing worst (58.6%). When knowledge of equivalence of one covariate across cells was added to the best guesser’s knowledge (Correct Guess 3), the differences across the procedures further narrowed (Simple randomization = 50.0%, MTI2 = 51.3%) Table 5.

**Table 5:** Proportion of Correct Guesses

	Correct Guess Rule #1	Correct Guess Rule #2	Correct Guess Rule #3
Attempt to guess:	cell (1 of 16)	Factor 1 (1 of 2 levels)	Factor 1 (1 of 2 levels)
Based on:	sample size of cell	sample size of level	sample size of level + covariate
SR	6.2%	50.1%	50.0%
SPB	10.0%	52.6%	50.2%
MTI2	19.2%	58.6%	51.3%
MTI3	18.2%	55.8%	51.1%
MSB	16.5%	51.1%	50.3%
Min	18.0%	57.5%	50.8%

Regarding cost and complexity, the study team’s primary question was whether the benefit of an alternative procedure to stratification with permuted blocks, which was the first procedure under consideration due to precedent [20] and past experiences of the research team, was worth the added cost of implementation. In our case, this cost required creation and ongoing use of a web-based form to determine study participant assignment. Such a form would require functionality not available in our primary database (RedCap), and thus a customized solution. Given that a co-investigator on the study had the coding skills and content knowledge to create such a form, it was decided that if one procedure stood out as superior, then it was worth using this co-investigator’s time for these purposes.

## Discussion

In step 3, the research team agreed that while simple randomization was the clear winner on unpredictability, the need for balance and equivalence across the sixteen cells required a different procedure. Regarding cell size balance, MTI2 and minimization performed best, with an average difference of 2.9 and 1.9 participants between the smallest and largest cells, respectively. Given the study team’s preference for sample imbalance no greater than 2, these findings resulted in a preference for one of these two methods. In regard to equivalence, minimization was the only procedure that resulted in no statistically significant differences of covariates assigned across cells. Although the minimization procedure resulted in 34% deterministic allocations, it performed similarly to other procedures on the proportion of correct guesses. Overall, likelihood of guessing correctly based on varying levels of knowledge did not vary greatly across the various procedures (e.g., range of correct factor guesses with knowledge of level sample size and covariate ranged from 50 to 51.3%).

Despite controversy regarding the use of minimization in randomized trials [22, 27, 28], the research team decided that the minimization procedure with a random element was a methodologically sound option for their MOST optimization trial that used a factorial experimental design. The team cited several reasons to support this decision. First, the large number of cells that are possible in a factorial experiment increases complexity and creates opportunities to easily incorporate randomness into the minimization algorithm (i.e., by breaking ties), thus at least partially countering the primary critique that minimization is completely deterministic. In our case, we also found that minimization was most successful in achieving the combination of both balance and equivalence across a large number of covariates, while maintaining an acceptable probability of guessing correctly with varying levels of knowledge considered in the guess. It is important to note that the research team was satisfied with a rate of 34% deterministic allocations both because of the correct guess results and because of the degree of equipoise inherent in their study design, which offers little reason to favor any one cell over another. Such a trade-off may not be acceptable in the context of other research studies.

Factorial design researchers who are less concerned with the possibility of significantly different levels of a few covariates, and more concerned with the proportion of deterministic allocations, may reasonably opt for an MTI procedure or stratification with permuted blocks based on our findings. Lastly, minimization, along with MTI and MSB, proved more difficult than the other procedures to implement in practice.

Notably, the advantage of minimization over MTI with respect to equivalence across covariates was observed in a limited set of variables, all of which were included in the minimization procedure. We did not consider equivalence on covariates not included in the minimization procedure, so the relative advantage of MTI versus minimization in this regard lies beyond the scope of this paper. Therefore, our results are restricted to cases where there is only a limited set of known covariates.

For researchers who are planning MOST studies with factorial designs, we strongly recommend use of computer simulation to test for the optimal randomization procedure in the study context, with consideration of more novel methods including MTI, MSB, and minimization with a randomization element for subject assignment. In doing so, we suggest following the process outlined here: reviewing and defining assignment procedures, conducting simulations that estimate the performance of each procedures in the context of the study, and reviewing results with the study team to decide upon the optimal method. In addition to providing head-to-head comparisons among methods, simulations offer the opportunity to refine algorithms by reweighting variables and/or introducing randomness to assignments and then evaluating results.

We note several limitations to the method we propose. Foremost, the predictive value of simulation results is only as good as the data on which the simulations are performed. If datasets used for simulations differ markedly from those collected prospectively, then results may differ as well. Moreover, although simulations can generate valuable evidence with regard to three different outcomes (i.e., randomness, balance, and equivalence), researchers are still presented with a multi-attribute decision problem. If a researcher anticipates that one method will be superior to the others on all outcomes for a study, the most rational choice for this study will be clear. However, in cases such as ours, in which minimization is superior with respect to some attributes (e.g., equivalence and balance) but inferior on others (e.g., unpredictability and anticipated cost/complexity), then the best decision will depend on the relative values researchers place on one attribute versus another.

## Conclusions

The MOST framework is gaining increased popularity and utility to refine multi-component clinical interventions [29]. MOST optimization trials will often include testing of multiple intervention components, require the use of factorial design, and present researchers with challenges when deciding the most appropriate subject allocation method. Our findings suggest that minimization with a random element to break ties is a promising subject assignment strategy for MOST optimization trials because it may result in optimally-balanced sample sizes and covariates across conditions, while also incorporating unpredictability with elements of randomization. Furthermore, computer simulations offer a valuable method for informing choices among the alternatives of subject assignment procedures.

## Data Availability

The datasets used and/or analyses during the current study are available from the corresponding author on reasonable request.

## Abbreviations

CONSORT: Consolidated Standards of Reporting Trials
MOST: Multiphase Optimization Strategy
RCT: Randomized Controlled Trial
